# Unusual bidirectional frequency dependence of dynamical susceptibility in hexagonal intermetallic Pr_2_Ni_0.95_Si_2.95_

**DOI:** 10.1038/s41598-018-32740-4

**Published:** 2018-10-05

**Authors:** Santanu Pakhira, Chandan Mazumdar, Abhik Basu, R. Ranganathan, R. N. Bhowmik, Biswarup Satpati

**Affiliations:** 1 Condensed Matter Physics Division, Saha Institute of Nuclear Physics, 1/AF, Bidhannagar, Kolkata, 700 064 India; 2 Department of Physics, Pondicherry University, R. V. Nagar, Kalapet, Pondicherry, 605014 India; 3 Surface Physics & Material Science Division, Saha Institute of Nuclear Physics, 1/AF, Bidhannagar, Kolkata, 700 064 India

## Abstract

In this study, the synthesis of a novel ternary intermetallic compound Pr_2_Ni_0.95_Si_2.95_ forming in single phase only by deliberately introducing vacancies in Ni/Si site is reported. The detailed studies on dc magnetization, heat capacity, ac magnetization & associated dynamical scaling, different types of non-equilibrium dynamical behaviour, *viz*., magnetic relaxation behaviour as a function of wait time and temperature, aging phenomena, and magnetic memory effect firmly establish that the compound exhibits spin freezing behaviour below 3.3 K (T_f_). However, below T_f_, temperature dependence of ac susceptibility data exhibit an additional peak that shows reverse frequency dependence to that generally observed in a glassy system. The unusual bidirectional frequency dependence in a single magnetic system is of significant interest and rarely reported in literature. Competing exchange interaction arising from *c*/*a* ~ 1 and crystallographic randomness driven magnetic phase separation has been argued to be responsible for such observation. The reverse frequency shift of the low temperature peak has been described on the basis of a simple phenomenological model proposed in this work.

## Introduction

A correlated magnetic spin system is called magnetically frustrated when it is unable to simultaneously minimize all its interaction energies between it’s ground state configuration of spins. Magnetically frustrated systems are rich in physics and one of the central themes in condensed-matter physics research not only from fundamental point of view, but also for application purposes. Frustration can have different origins in a magnetic system, *e.g*., favourable lattice geometry, competing magnetic interactions. In the presence of disorder such frustrated systems may often drive to a spin freezing state below a typical temperature, called spin freezing temperature. In these systems, presence of random magnetic anisotropy often offer a breeding ground for several unconventional physical properties^1–7^.

**Figure 1:**
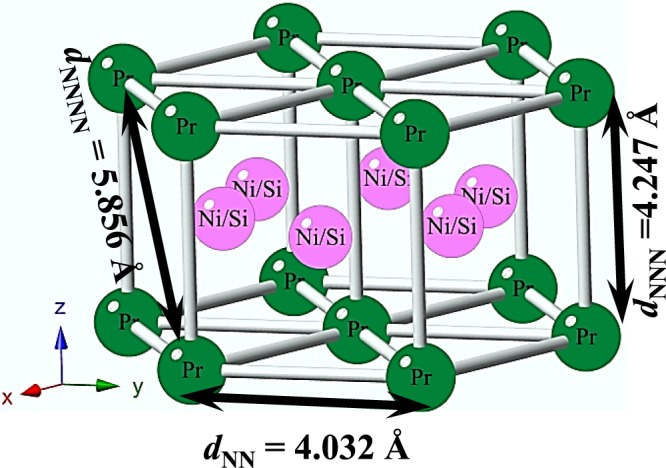
Crystal structure of Pr_2_Ni_0.95_Si_2.95_.

During the last few years, ternary silicide intermetallic compounds of *R*_2_TSi_3_ type, with *R* = rare-earth element and T = transition metal, have attracted much attention due to their highly interesting magnetic properties. Most of the *R*_2_TSi_3_ type of compounds crystallizes in hexagonal AlB_2_ type crystal structure with space group *P6/mmm*^8^. The rare-earth (*R*) ions sit on the Al site symmetry (Wyckoff *1a*) forming triangles of nearest neighbour, while the T and Si atoms are randomly distributed (Wyckoff *2d*) between two hexagonal layers (made by edge sharing triangles of rare-earths). Thus, the crystal structure consists of hexagonal rare-earth atoms layer and randomly distributed T-Si layer along the hexagonal *c*-axis (Fig. 1). In these type of compounds, geometrical frustration^1,9,10^ can arises due to the presence of antiferromagnetic interaction in the hexagonal crystal structure between *R* ions. Additionally, in *R*_2_TSi_3_ type of compounds, the lattice parameters *a* and *c* values are comparable yielding *c*/*a* ~ 1, *i.e*., nearest-neighbour (NN) and next-nearest-neighbour (NNN) exchange interactions (J) are comparable and a strong frustration occurs when J_NN_ and J_NNN_ are of opposite sign. Quite a few members of this type exhibit magnetically frustrated spin-glass behaviour due to competing magnetic interactions^5,11–15^. On the other hand T and Si ions are randomly distributed between the two hexagonal layers comprising rare-earth ions, that causes variation in local environment between the rare-earth ions. This type of variation of the electronic environment between the rare earth ions are favourable for the coexistence of magnetically different phases in a crystallographically single phase compound. A considerable number of such magnetic phase separated systems exhibit glassy behaviour. The glassiness in such a phase-separated system can have two likely origins: first, the slow dynamics of the coexisting phases due to their metastability, and secondly, the frustration arising from the magnetic interaction between two clusters having a distinctly different magnetic nature.

Among the *R*_2_NiSi_3_ family, it is reported that Gd_2_NiSi_3_ and Er_2_NiSi_3_ exhibit spin glass state coupled with AFM type ordering^5^. However, Ho_2_Ni_0.95_Si_2.95_ is a highly frustrated magnetic system consisting of very weakly correlated FM clusters, the correlation of which extends up to only 10–12 unit cells^6^. The correlation between the FM clusters is so weak that any glassy state formation is forbidden in this system. In this present work, we report the successful synthesis of a novel intermetallic compound Pr_2_Ni_0.95_Si_2.95_, which form in single phase only with defect crystal structure. The compound undergoes spin cluster glass behaviour below 3.3 K. The main interest in this material stems from the fact that unusual coexistence of opposite frequency shift of the transition temperatures has been observed for this compound, which is rather hard to find in nature. Randomness driven phase segregation has been argued to be responsible for such unique observation. A simple phenomenological model is proposed that reproduces the main features of this unexpected observation qualitatively.

## Results and Discussion

**Figure 2:**
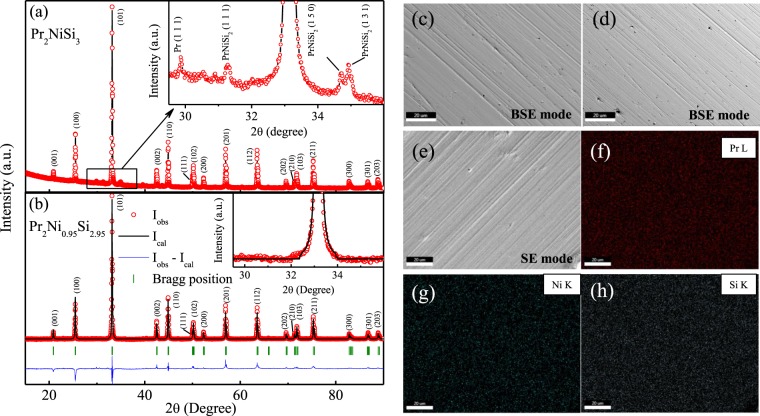
Room temperature powder XRD pattern of (**a**) Pr_2_NiSi_3_ and (**b**) Pr_2_Ni_0.95_Si_2.95_ along with full Rietveld refinement. Inset shows the presence of secondary phases of Pr and PrNiSi_2_ type in Pr_2_NiSi_3_ (upper), while Pr_2_Ni_0.95_Si_2.95_ (lower) form in single phase. SEM image in (**c**,**d**) BSE mode in two different regions and in (**e**) SE mode (tomography). (**f**–**h**) Elemental mapping by SEM equipped with EDX on the surface of Pr_2_Ni_0.95_Si_2.95_ from the region presented in (**e**).

**Figure 3:**
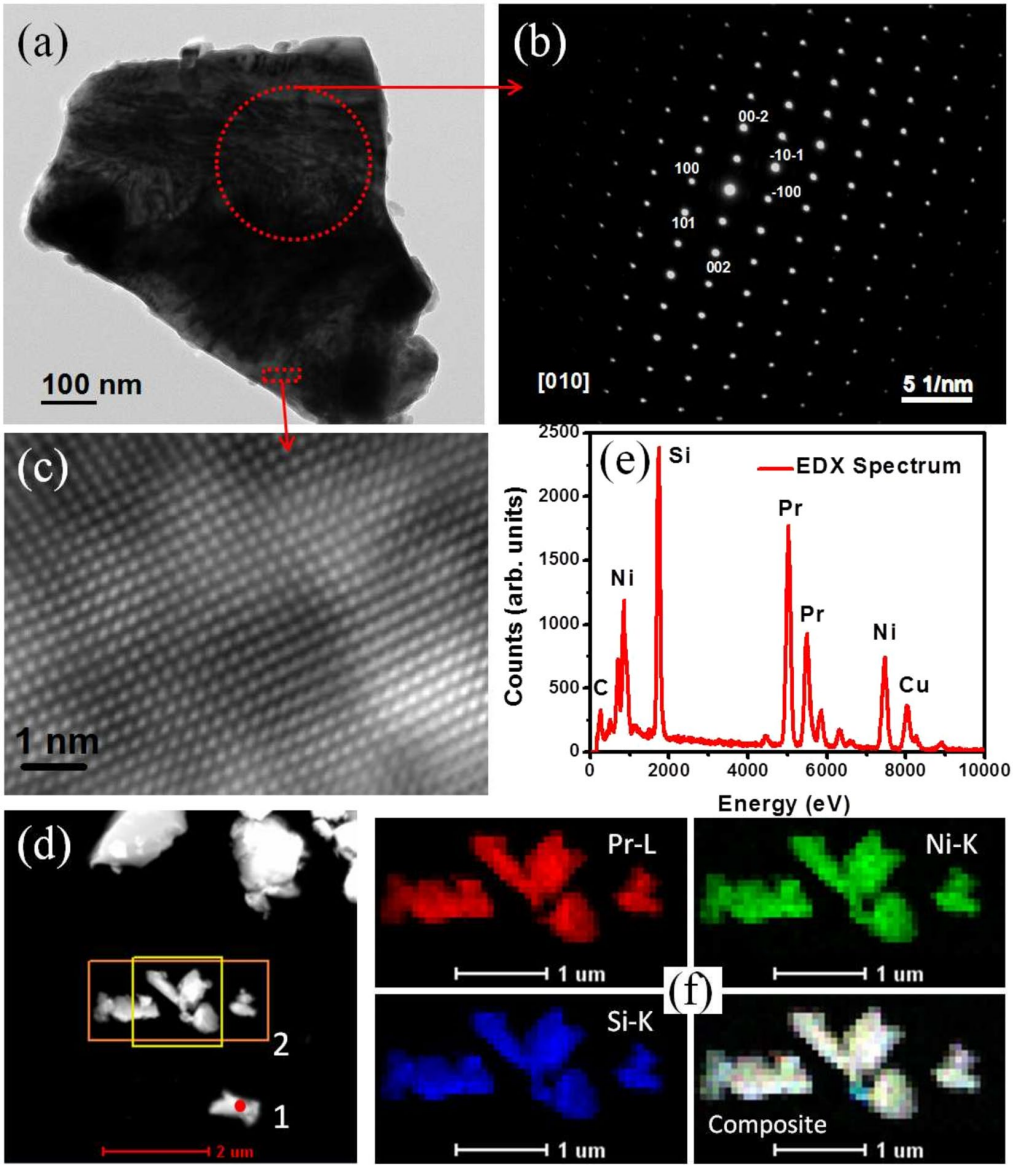
(**a**) Low magnification bright-field TEM image, (**b**) Selected area electron diffraction pattern from the circular area in (**a**), (**c**) High resolution TEM image from the rectangular area in (**a**), (**d**) STEM-HAADF image, (**e**) EDX spectrum from area 1 in (**e**), (**f**) EDX elemental maps and overlay of different elements.

The room temperature XRD pattern of the stoichiometric Pr_2_NiSi_3_ is shown in Fig. 2(a). As seen from the figure, around the (101) reflection of Pr_2_NiSi_3_ phase, minor contributions of additional phases can also be observed (~6% of main phase). On the basis of detailed analysis using PDXL software package, it is determined that the additional phases belong to PrNiSi_2_ type of stoichiometry in addition to another minor contribution from Pr metal. It may be noted here that although many similar *R*_2_TX_3_ type of compounds have been reported in literature to form along with a secondary phase which were of *R*T_2_X_2_ type^16^, in contrast to PrNiSi_2_ found in our system. We had earlier reported similar RNiSi_2_ type of secondary phase in *R*_2_NiSi_3_ (*R* = Ho, Tm) compounds^6,17^. These additional XRD peaks, however, can not be removed by thermal annealing as the single phase compounds could be synthesized only by deliberately tweaking the proportion of initial stoichiometry by subtracting the volume fraction of elements present in the secondary phase. After the first attempt, the volume fraction of the secondary phase got drastically reduced and continuing similar steps iteratively single phase XRD pattern is finally achieved. The single phase compound thus could be synthesized only by introducing defects in Ni and Si sites having the starting composition Pr_2_Ni_0.95_Si_2.95_ where all the peaks in the XRD pattern can be well indexed with space group *P6/mmm* [Fig. 2(b)]. The refined stoichiometry from the Rietveld refinement using FULLPROF software^18^ agrees well with the starting composition of the compound. The different crystallographic parameters estimated from the full-Rietveld analysis are summarized in Table 1. One may find that, *c*/*a* $\simeq$ 1.05, suggesting that NN and NNN distances between the magnetic Pr ions are close enough. Furthermore, the scanning electron microscopy (SEM) measurements were carried out in both Back-scatter electron detector (BSE) mode and secondary electrons (SE) mode (tomographic mode) to check the homogeneity of the sample (Fig. 2(c–e)). The uniform chemical contrast of BSE signal confirms the phase homogeneity of the sample. The SEM and energy dispersive X-ray spectroscopy (EDX) reveals an essentially single phase nature of the compound with average composition Pr_2_Ni_0.89_Si_2.99_ that is in good agreement with that obtained from Rietveld refinement (Fig. 2(c–h)). Transmission Electron Microscopy (TEM) was also carried out to investigate the nature of phase, composition and crystallinity of intermetallic compound Pr_2_Ni_0.95_Si_2.95_. Figure 3(a) represents the TEM image of one grain of the compound. The selected area electron diffraction (SAED) pattern from a region marked by a dotted circle is shown in Fig. 3(b), which indicates the single crystalline nature of the material. Measurement was carried out on several grains to confirm the single crystalline nature. SAED was indexed using the lattice parameters of hexagonal crystal structure (lattice parameters, *a* = 4.032 Å and *c* = 4.247 Å). Though some of the earlier reports yield superstructure formation in single crystalline *R*_2_TSi_3_ type of materials^19^, no evidence of any such superstructure formation could be traced from the electron diffraction pattern in the defect structure compound Pr_2_Ni_0.95_Si_2.95_ [Fig. 3(b)]. The HRTEM image in Fig. 3(c) from a dotted red box region again clearly showing the single crystalline phase. To investigate the chemical composition of the compound, we have performed energy dispersive X-ray spectroscopy (EDX) in high-angle annular dark field (HAADF) scanning transmission electron microscopy (STEM-HAADF) mode. Figure 3(d) shows the STEM-HAADF image, and Fig. 3(e) showing EDX spectrum indicating the presence of Pr, Ni and Si. The observed Cu and C signal is due to the C-coated Cu grid. For a detailed distribution of atomic content inside the nanocrystals, elemental mapping of Pr, Ni and Si was performed using drift corrected EDX spectrum imaging using STEM-HAADF mode. The STEM-HAADF image in Fig. 3(d) and the corresponding chemical maps from orange boxed region for Pr, Ni and Si acquired using Pr-L, Ni-K and Si-K energy and overlay of three images are presented in Fig. 3(f), respectively. EDX maps were collected while beam of about 2 nm was scanning across the marked area. In this process X-rays were collected point by point from 50 points in x-direction and 25 points in y-direction from the STEM-HAADF image shown in Fig. 3(d). Composite maps of Pr, Ni and Si were presented in Fig. 3(f) confirm the single phase nature of the material with stoichiometry Pr_2_Ni_0.97_Si_2.89_.

**Table 1:** The estimated crystallographic parameters from the full-Rietveld analysis of powder x-ray diffraction data of Pr_2_Ni_0.95_Si_2.95_ taken at room temperature.

Compound		Pr_2_Ni_0:95_Si_2:95_				
Space group		*P6/mmm*, (No. 191)				
Lattice parameters						
*a*(Å)		4.032				
*c*(Å)		4.247				
Cell Volume, V_cell_ (Å^3^)		59.831(3)				
*R*_*f*_		6.8				
*R*_*Bragg*_		9.2				
**Atomic positions**						
**Atom**	**Wyckoff Symbol**	***x***	***y***	***z***	occ. parameter*	**site occupancy** × **100%**
Pr	1*a*	0	0	0	0.0416	100
Ni	2*d*	1/3	2/3	1/2	0.0197(1)	23.65 (10)
Si	2*d*	1/3	2/3	1/2	0.0614(1)	73.68 (10)

*occ. parameter = site multiplicity/highest multiplicity of space group.

**Figure 4:**
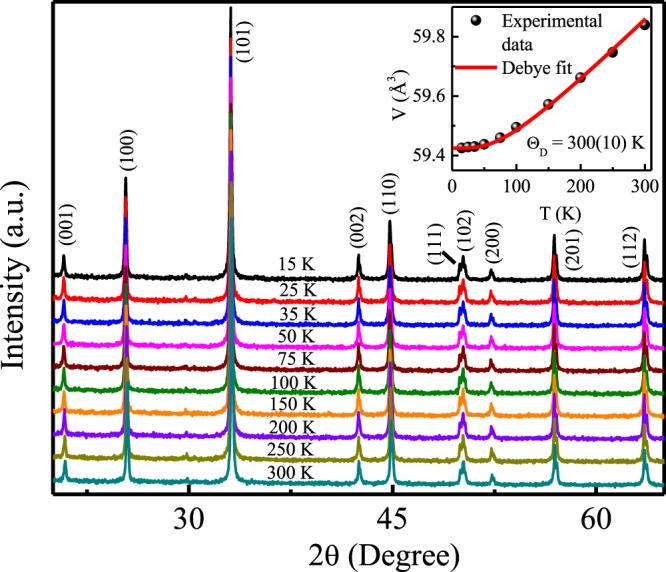
Observed powder XRD profiles of Pr_2_Ni_0.95_Si_2.95_ measured at various temperatures between 15 and 300 K. Inset shows the temperature evolution of the unit-cell volume along with fit to Eq. 1. Error bars are within the symbol size limit.

The low temperature XRD measurements have also been carried out for the synthesized compound to investigate the evolution of crystal structure with temperature. Figure 4 shows the XRD patterns measured at different temperatures in the temperature region 15–300 K for Pr_2_Ni_0.95_Si_2.95_. The low temperature XRD patterns reveal that crystal structure remains conserved down to 15 K. The temperature evolution of the estimated unit cell volume has been analysed using the relation,

1 $$V(T)=\gamma U(T)/{K}_{0}+{V}_{0}$$

where, *V*_0_ is the unit cell volume at zero temperature limit, *K*_0_ is the bulk modulus of the system and *γ* is the Grüneisen parameter. According to Dbye approximation, the internal energy, *U*(*T*), can be expressed as,

2 $$U(T)=9N{k}_{B}T{(\frac{T}{{{\rm{\Theta }}}_{D}})}^{3}\,{\int }_{0}^{\frac{{{\rm{\Theta }}}_{D}}{T}}\,\frac{{x}^{3}}{{e}^{x}-1}dx$$

with *N* is the total number of atoms per unit cell and $x=\frac{\hslash \omega }{{k}_{B}T}$. Using this approximation, Debye temperatures for the compound found to be 300 ± 10 K, which suggests quite a strong spin-orbit interaction (higher phonon energy mode) in the system (inset: Fig. 4). This value of Θ_*D*_ founds to be similar to that estimated for some other rare-earth analogues of the same family^5^.

**Figure 5:**
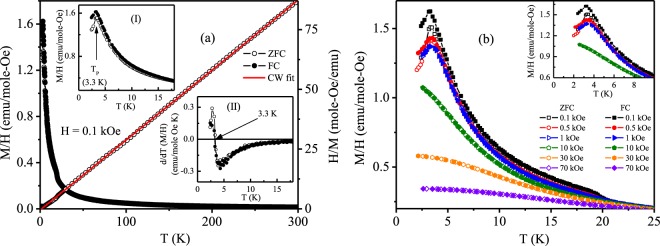
(**a**) ZFC and FC dc magnetization (M/H) (left panel) and inverse ZFC magnetic susceptibility (H/M) (right panel) as a function of temperature for Pr_2_Ni_0.95_Si_2.95_ at an applied magnetic field of 0.1 kOe. Inset (I): expanded low temperature region of ZFC and FC magnetic susceptibility. Inset (II) shows temperature derivative of ZFC and FC susceptibility as a function of temperature. (**b**) M/H(T) for different applied magnetic fields. Inset shows expanded low temperature region.

The dc magnetic susceptibility (M/H) as a function of temperature measured in zero-field-cooled (ZFC) and field-cooled (FC) modes for Pr_2_Ni_0.95_Si_2.95_ under a field of 0.1 kOe are shown in Fig. 5(a). In both the measurements, a maxima is observed at the peak temperature T_P_ = 3.3 K. Temperature derivative of the magnetization change their sign at the peak temperature, which is commonly considered as a signature of antiferromagnetic ordering [Inset (II): Fig. 5(a)]. At low temperature, *χ*_ZFC_(T) and *χ*_FC_(T) bifurcates from each other suggesting the presence of irreversibility (magnetic metastability) in the system^20^. At higher applied field strength, the extent of bifurcation decreases and the peak becomes broader. For applied field H ≥ 10 kOe, *χ*_ZFC_(T) and *χ*_FC_(T) coincide with each other and the peak disappears with the exhibition of ferromagnetic type behaviour [Fig. 5(b)]. The temperature dependence of inverse susceptibility deviates from linear Curie-Weiss (CW) behaviour below 35 K, either due to the presence of crystalline electric field (CEF) effect or the presence of short range magnetic correlations up to such higher temperature, which is almost 10 times higher than the peak temperature [right panel: Fig. 5(a)]. The CW fit of inverse susceptibility in the temperature region 35–300 K yield a positive paramagnetic Curie-Weiss temperature, *θ*_*p*_ = 0.5 K, suggesting the presence of considerable ferromagnetic interaction in this system. The calculated paramagnetic effective moment (*μ*_eff_) is found to be 3.66 *μ*_B_/Pr-ion which is close to theoretical paramagnetic free moment value of Pr^3+^ ion (3.58 *μ*_B_).

**Figure 6:**
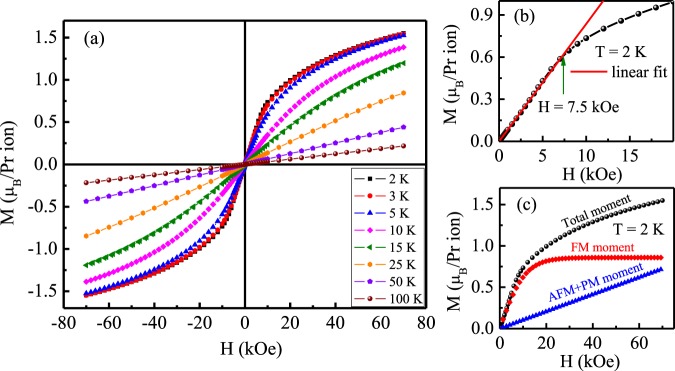
(**a**) Field dependence of isothermal magnetization for Pr_2_Ni_0.95_Si_2.95_ at different temperatures. (**b**) M(H) at T = 2 K along with linear fitted region. (**c**) M(H) at T = 2 K along with extracted different types of magnetic volume fraction using Eq. 3.

The field dependence of isothermal magnetization at different temperatures are shown in Fig. 6(a). The M(H) behaviour at the lowest temperature (2 K) is linear upto applied magnetic field of 7.5 kOe [Fig. 6(b)], while at higher applied field it tends to saturate. Despite the application of a strong magnetic field of 70 kOe, full saturation behaviour of the magnetization could not be obtained. The value of the magnetic moment at 2 K for 70 kOe applied field is 1.55 *μ*_B_/Pr-ion, which is less than the expected saturation moment value for parallel spin configuration of Pr^3+^ (*gJ* = 3.2 *μ*_B_, with *g* = 4/5, *J* = 4) moments. No magnetic hysteresis is observed down to 2 K. The M(H) is not linear even at T = 25 K, which is in the same line with the observation that the inverse susceptibility deviates from linear behaviour below 35 K. At further higher temperatures, however, M(H) behaviour found to be linear, similar to that expected in a paramagnetic system. The field dependence of magnetic isotherms can be analysed considering a combined effect of ferromagnetic and antiferromagnetic and(or) paramagnetic isotherms by the equation,

3 $$M(H)=A[\,\tanh (BH)]+CH$$

where *A*, *B* and *C* are fitting parameters. A significant amount of ferromagnetic moment volume fraction is evidenced along with linear (combined antiferromagnetic and paramagnetic) moment volume fraction for the compound [Fig. 6(c)].

**Figure 7:**
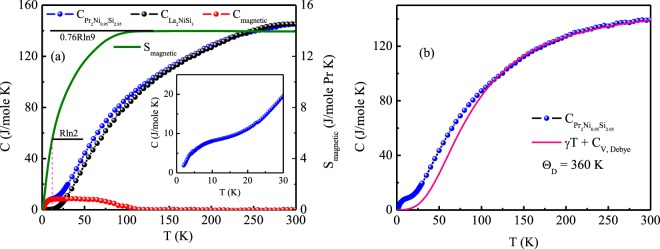
(**a**) Zero field heat capacity of Pr_2_Ni_0.95_Si_2.95_, La_2_NiSi_3_, magnetic contribution of heat capacity (left panel) and magnetic entropy (right panel) as a function of temperature. Inset shows expanded low temperature region of heat capacity for Pr_2_Ni_0.95_Si_2.95_. (**b**) Heat capacity data is fitted to Debye model according to Eq. 4.

The specific heat data measured under relaxation technique of Pr_2_Ni_0.95_Si_2.95_ and the nonmagnetic reference compound La_2_NiSi_3_ are shown in Fig. 7(a). Corresponding to the peak (T_P_) in dc magnetic susceptibility data, we do not observe any sharp anomaly near 3.3 K that can indicate a magnetic phase transition. Instead, C(T) of Pr_2_Ni_0.95_Si_2.95_ shows a very broad anomaly in the temperature range 2–15 K. The absence of sharp peak around T_P_ = 3.3 K suggest the possible absence of any long range AFM order, that was observed earlier in some other members of this family^5^. Further, we have found that the specific heat attains a saturation value (145.5 J/mole-K) at room temperature which is close to the expected classical Dulong-Petit value of C = 3 *n*R = 17.7 R = 147.1 J/mole-K for this compound. The magnetic contribution (C_magnetic_) of heat capacity also shows a broad hump over a quite wide temperature range, which may be ascribed to either Schottky-type anomaly originating from crystalline electric field (CEF) or the presence of short range magnetic interaction in this temperature range. The calculated magnetic entropy value at T_P_ (= 3.3 K) reaches 0.17 R J/mole-Pr-K which is only about 24% of R*ln*2, where R*ln*2 being the minimum entropy required for a spin doublet ground state. The estimated magnetic entropy thus appears to be rather small for the occurrence of any true long-range magnetic ordering around the phase transition temperature, T_P_. The magnetic entropy saturates at much higher temperature (T > 100 K) with the value 13.95 J/mole-Pr-K (0.76 R*ln*9), which is lower than that expected for trivalent Pr-ion [R*ln*(*2J* + *1*) = R*ln*9, with *J* = 4]. This may be due to CEF effect or presence of short-range magnetic correlations up to such higher temperature. In the temperature range 110–300 K, the specific heat behaviour can be fitted by

4 $$C(T)=\gamma T+n{C}_{V,Debye}(T)$$

where, the coefficient *γ* is usually due to the electronic contribution (Sommerfeld coefficient), *n* is the number of atoms per formula unit (here we have taken *n* = 5.9) and C_*V*,*Debye*_ is the lattice heat capacity contribution owing to acoustic phonon at constant volume. Debye heat capacity per mole is expressed as,

5 $${C}_{V,Debye}=9R{(\frac{T}{{{\rm{\Theta }}}_{D}})}^{3}\,{\int }_{0}^{{{\rm{\Theta }}}_{D}/T}\,\frac{{x}^{4}{e}^{x}}{{({e}^{x}-1)}^{2}}dx$$

This is an accurate analytic Padé approximation function of T/Θ_*D*_. The estimated Debye temperature (Θ_*D*_) founds to be 360 K [Fig. 7(b)], which is slightly higher to that estimated from the temperature evolution of unit cell volume data. The low temperature mismatch of the fit arises due to the CEF contribution in addition to possible short range magnetic correlations, which are not taken care by the above analysis of specific heat.

**Figure 8:**
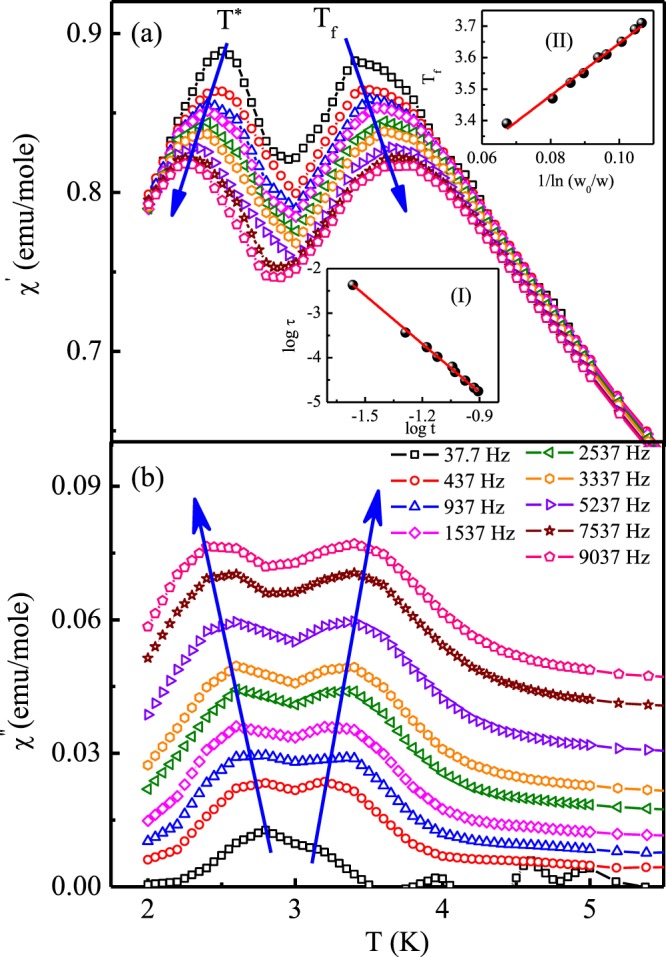
(**a**) The real part of ac magnetic susceptibility of Pr_2_Ni_0.95_Si_2.95_ as a function of temperature at different frequencies. Inset (I) shows the critical slowing down behaviour of freezing temperature, where t = (T_f_ − T_SG_/T_SG_). Inset (II) shows the variation of freezing temperature, T_f_, as a function of ln(*ν*_0_/*ν*). The solid line shows the fit to Vogel-Fulcher law. (**b**) The imaginary part of ac magnetic susceptibility of Pr_2_Ni_0.95_Si_2.95_ as a function of temperature at different frequencies.

Since short range magnetic correlations can be probed through most sensitive technique of ac susceptibility measurement^21^, we have carried out dynamical susceptibility measurements. The temperature dependence of real part (*χ*′) and imaginary part (*χ*″) of ac susceptibility at different frequencies for Pr_2_Ni_0.95_Si_2.95_ is shown in Fig. 8(a,b), respectively. As seen from the figure, close to the peak temperature (T_P_ = 3.3 K) determined from dc magnetization data, a peak is observed that is very sensitive to applied frequency. The peak temperature shifts to higher temperature region with increase in ac frequency (3.39 K for *ν* = 37.7 Hz and 3.71 K for *ν* = 9037 Hz). This positive shift in the peak temperature with increasing frequency reveals glassy or superparamagnetic state formation in Pr_2_Ni_0.95_Si_2.95_.

In a typical glassy system, the frequency dependence of peak shift is generally classified by the parameter^2^,

6 $$\delta {T}_{f}=\frac{{\rm{\Delta }}{T}_{f}}{{T}_{f}\,{\rm{\Delta }}\,{\mathrm{log}}_{10}\,\nu }$$

The observed values of *δT*_*f*_ for canonical spin glasses are found to be ~10^−3^. However, in case of different spin cluster glass compound, *δT*_*f*_ is of the order of 10^−2^ and for several reported superparamagnetic systems *δT*_*f*_ ~ 10^−1^. We have found *δT*_*f*_ = 0.031 for Pr_2_Ni_0.95_Si_2.95_, which is in the range of that for different reported cluster glass systems. Thus, frequency dependent ac susceptibility measurement signifies glassy state formation in Pr_2_Ni_0.95_Si_2.95_ with spin freezing temperature, T_f_ = 3.3 K.

The frequency dependence of freezing temperature (T_f_) was analyzed using the concepts of the dynamic scaling theory that predicts the critical power law of the form^2,22^,

7 $$\tau ={\tau }_{0}{(\frac{{T}_{f}-{T}_{SG}}{{T}_{SG}})}^{-z\nu ^{\prime} }$$

where *τ* (=1/*ν*) is the relaxation time corresponding to the excitation frequency, *τ*_0_ is the characteristic relaxation time of single spin flip, T_SG_ is the spin glass temperature at zero frequency and the dynamic critical exponent is *zν*′ [where, *ν*′ is the critical exponent of correlation length, *ξ* = (*T*_*f*_/*T*_*SG*_ − 1)^−*ν*′^ and the dynamical scaling relates *τ* to *ξ* as *τ* ~ *ξ*^*z*^]. For a spin glass system, *zν*′ typically lies between 4 and 12. The value of *τ*_0_ for canonical spin glasses fall in the characteristic range ~10^−12^–10^−13^ s^23^, whereas, for a spin cluster glass system *τ*_0_ value typically founds to be in the range ~10^−4^–10^−11^ s^24^. Relatively higher values of *τ*_0_ correspond to superparamagnetic state formation. The linear fit of log − log plot of *τ* as a function of reduced temperature, *t*, yields ${\tau }_{0}\simeq {10}^{-8}$s and *zν*′ = 4.02 for Pr_2_Ni_0.95_Si_2.95_ [inset (I) of Fig. 8(a)]. Thus spin freezing behaviour in this compound can be described by cluster glass state formation, where rather than individual spins, clusters of spins take part in the freezing process.

In glassy systems, spin dynamics near the freezing temperature can be modeled by another dynamical scaling law, known as the empirical Vogel-Fulcher relation^2,25^, described as,

8 $$\nu ={\nu }_{0}\,\exp \,(\,-\,\frac{{E}_{a}}{{K}_{B}({T}_{f}-{T}_{0})})$$

where the fitting parameters *ν*_0_, *E*_*a*_ and T_0_ are known as characteristic frequency, activation energy and the Vogel-Fulcher temperature, respectively. For canonical spin glasses the ratio, $\frac{{E}_{a}}{{K}_{B}}/{T}_{0}$ generally found to be close to 1, and the relatively larger value corresponds to while for spin cluster glass state formation. The best estimated values obtained for Pr_2_Ni_0.95_Si_2.95_ are $\frac{{E}_{a}}{{K}_{B}}=8.31$ K and T_0_ = 2.81 K [inset (II) of Fig. 8(a)]. This result also indicates cluster glass state formation for this compound. Thus on the basis of all the results obtained from dynamical scaling laws of ac susceptibility data, it is argued that the newly synthesized compound Pr_2_Ni_0.95_Si_2.95_ exhibits spin cluster glass behaviour below its freezing temperature (3.3 K).

The most interesting part of the ac susceptibility measurement for Pr_2_Ni_0.95_Si_2.95_ is the observation of a distinct, additional peak (say, at T = T*) slightly below the spin freezing temperature, T_f_. The observation of the second peak at low temperature might suggest the presence of an additional magnetic phase in this compound. Earlier, we have reported that the magnetic ground state of isostructural Er_2_NiSi_3_ compound exhibits two different antiferromagnetically ordered phases in addition to the presence of a spin glass phase^5^. Isostructural compound Sm_2_Ni_0.87_Si_2.87_ exhibits magnetically frustrated cluster glass behaviour in the presence of macroscopically different competing magnetic phases^13^. It may be noted here that the magnetic phase inhomogeneity in these materials arises due to the local environmental variation among the rare-earth ions mediated by non-magnetic ions, although the compound is considered to be crystallographically single phase as determined from XRD and neutron diffraction measurements. This type of inhomogeneity produces random magnetic anisotropy (RMA) in these material, which is responsible for different magnetic phases coexistence with limited correlation length. Depending on the correlation length these type of systems exhibit spatially limited magnetic ordering coupled with glassy magnetic phases. Accordingly, it appears that the two peaks in ac susceptibility measurements of Pr_2_Ni_0.95_Si_2.95_ might arise due to the formation of two different magnetic phases in crystallographically single phase material.

From Fig. 8, one can notice that similar to the frequency dependent high temperature peak (T_f_) shift due to the glassy behaviour, the low temperature peak also exhibit a frequency dependent shift in temperature that apparently exclude the possibility of its origin due to long range order. However, surprisingly, the frequency dependence of the low temperature peak appear to be in the reverse direction than that expected in case of glassy transitions. This is a highly unusual behaviour, and only a very few oxide compounds have been reported to exhibit such behaviour in literatures^26–29^. However, it may be noted here that so far, only one work has been reported in the literature with bidirectional frequency dependence, that too on an oxide compound NaNiO_2_ which follows superexchange mechanism^26^. In contrast, Pr_2_Ni_0.95_Si_2.95_ is an intermetallic compound, where the magnetic exchange interaction is of RKKY-type. Furthermore, despite the superficial similarity between the phenomenon reported in NaNiO_2_ and in our work on Pr_2_Ni_0.95_Si_2.95_, there are significant differences. While in the oxide compound, the spin glass state has the freezing temperature lower than the temperature where the reverse frequency shift is observed, in our case the trend is just the opposite. It was first reported in NaNiO_2_, that exhibit a spin glass behaviour close to 3 K and undergoes antiferromagnetic ordering below 19.5 K (T_N_)^26^. However, slightly above the T_N_, the ac susceptibility measurement exhibit an additional peak around 24 K, whose frequency dependence is quite opposite to that observed glassy behaviour around 3 K. The muon decay asymmetry measurement in the temperature range 19.5–24 K could be modeled with two exponential components for NaNiO_2_, one having much faster relaxing rate than the other and also varying differently with temperature. Since the amplitude of the faster relaxing component decreases with increasing temperature, it was suggested that the magnetic precursor effect to the antiferromagnetic order at 19.5 K persists up to 24 K, with the gradual decrease of relative volume fraction of the slowly fluctuating magnetic cluster sizes. It has been argued that this temperature dependent change in volume fraction of these magnetic clusters of different relaxation times may be related to the apparently unconventional peak-shift in the reverse direction. All the subsequent observations of such reverse peak shift in ac susceptibility measurements primarily used the same argument. Similar argument may hold good in our case of Pr_2_Ni_0.95_Si_2.95_ as well.

However, there may be another mechanism that results in such reverse peak shift in ac susceptibility. The short range magnetic order due to the coalescing of magnetic spins takes place at temperature that is reflected in the peak in ac susceptibility. For a very small sizes of the clusters, the magnetic spins in a cluster favor parallel alignment along the externally applied magnetic field. As the external magnetic field strength decreases, the clustering process tends to shift towards lower temperature. Similarly, for a particular ac field, as the applied ac frequency increases, the average field in a particular direction over a fixed period of time would also decrease and therefore would have a similar effect as that of reduced field. Thus, the coalescing process of the magnetic clusters would gradually shift to lower temperature with increasing frequency, as is the phenomenon observed in our case of Pr_2_Ni_0.95_Si_2.95_. However, in such cases, very low strength of the applied ac field strength (4 Oe) suggest that, both the magnetic moment as well as sizes of the clusters have to be significantly small so that even such small ac field can have observable impact. It may be mentioned here that, we have earlier reported that the coherence length of the magnetically ordered phase of Er_2_NiSi_3_ at 0.8T_N_ ~ 143 Å (~35 unit cells) and tend to saturate at low temperature with the value of 250 Å (~60 unit cells)^5^. The long range magnetic ordering could not be seen in Ho_2_Ni_0.95_Si_2.95_ as the coherence length at 1.5 K is about 35 Å (~8 unit cells), although magnetization and heat capacity studies reveal magnetic clusters appear to form below 3.6 K^6^. The temperature dependence of ac susceptibility peak at 3.6 K remain insensitive to change in frequency, which further reveals magnetic clusters in Ho_2_Ni_0.95_Si_2.95_ even do not exhibit inter-cluster correlations, therefore no cluster-glass phenomenon could be seen either. On the other hand, in Sm_2_Ni_0.87_Si_2.87_ the ferromagnetic clusters are correlated to each other and give rise to cluster glass state formation below 6.6 K^13^. Since the ground state of R_2_NiSi_3_ series of compounds have been earlier shown to have comprised of multiple magnetic phase, *e.g*., different antiferromagnetic and glassy phases in Er_2_NiSi_3_, we propose that magnetic ground state of Pr_2_Ni_0.95_Si_2.95_ consists of at least two magnetic phases, a magnetic glassy phase coupled with of non-correlated small magnetic clusters. The origin of the coexistence of different magnetic phases lies in the crystal structure of the compound. In Pr_2_Ni_0.95_Si_2.95_, as *c*/*a* $\simeq$ 1.05, the nearest-neighbour exchange interaction (J_NN_) and next-nearest-neighbour exchange interaction (J_NNN_) are of comparable strength. As in these system, exchange interaction is RKKY type and the strength (J_ex_) of which depends on the interatomic distance (*d*) as, J_ex_ ∝ 1/*d*^3^, the third-nearest-neighbour exchange interaction strength is relatively insignificant. The dc magnetic measurement results further suggest that J_NN_ and J_NNN_ are of opposite sign, that causes a strong frustration in the system. Additionally, Ni and Si ions are inhomogeneously distributed between the hexagonal layers comprising of Pr ions and is responsible for the variation in the local electronic environment among the Pr-ions. In the presence of such strong frustration and crystallographic disorder, the magnetic ground state of Pr_2_Ni_0.95_Si_2.95_ founds to be magnetically inhomogeneous. We also believe that the size of the magnetic clusters in Pr_2_Ni_0.95_Si_2.95_ are of even further reduced length scale (smaller than 8 unit cells). Since the magnetic moment of Pr is much smaller (${\mu }_{{\rm{\Pr }}}\ll {\mu }_{{\rm{Ho}}},\,{\mu }_{{\rm{Er}}}$), the effect of frequency dependence of ac magnetic field may produce an effect described as above. Additionally, in case of Er_2_NiSi_3_, we have also shown that, for the antiferromagnetically ordered phase the propagation vectors (*k*) are quite small, leading to a very large magnetic full Bloch wave of about 6–10 unit cells. If the cluster size in Pr_2_Ni_0.95_Si_2.95_ is smaller than the Bloch wave length, the effective moments in the cluster act ferromagnetic like and as a result the coalescing temperature of the magnetic clusters will decrease with the increase of frequency, *i.e*., the decrease of effective field value. This type of substantial confinement of antiferromagnetic spin waves are reported to being sensitive as ferromagnetic order in Lanthanide metals. It may be mentioned here that the frequency dependent variation of RKKY interactions has already been investigated theoretically earlier.

While a full microscopic modeling of the observed reverse peak shift of the ac susceptibilities is beyond the scope of the present work, we now provide a simple phenomenological model that is based on the above-mentioned physical heuristic arguments and qualitatively reproduce the general experimental features. In fact, by using this model we argue that the *ω*-dependent overall vertical shift observed in the plots of the susceptibilities in Fig. 8 occurs even for a single isolated spin in an external magnetic field, where as the horizontal reverse shift of one of the peaks in the same plots is essentially consequences of interactions. Consider first a single spin *S* in an external magnetic field *h*(*t*). The simplest dynamical equation for *S* in the overdamped limit reads

9 $$\frac{dS}{dt}=-\,{\rm{\Gamma }}S+{\rm{\Gamma }}h(t),$$

with Γ, a constant, is the damping coefficient. This yields for the frequency *ω* dependent susceptibility *χ*(*ω*) as^30^

10 $$\chi (\omega )=\frac{{\rm{\Gamma }}}{-i\omega +{\rm{\Gamma }}},$$

that is in general a complex function of *ω*. The real part *χ*′(*ω*) of *χ*(*ω*) is thus given by

11 $$\chi ^{\prime} (\omega )=\frac{{{\rm{\Gamma }}}^{2}}{{\omega }^{2}+{{\rm{\Gamma }}}^{2}},$$

where as, the imaginary part *χ*″(*ω*) is

12 $$\chi ^{\prime\prime} (\omega )=\frac{\omega {\rm{\Gamma }}}{{\omega }^{2}+{{\rm{\Gamma }}}^{2}}.$$

In the limit of large damping (or, low *ω*), such that $\omega /{\rm{\Gamma }}\ll 1$, the leading order *ω*-dependences of *χ*′(*ω*) and *χ*″(*ω*) reads

13 $$\chi ^{\prime} (\omega )=1-\frac{{\omega }^{2}}{{{\rm{\Gamma }}}^{2}}+O({\omega }^{4}),$$

that decreases as *ω* increases for large damping. In contrast,

14 $$\chi ^{\prime\prime} (\omega )=\frac{\omega }{{\rm{\Gamma }}}+O({\omega }^{3})$$

rises with *ω* for large damping. Equations (13) and (14) then qualitatively reproduce the decrease and increase in *χ*′(*ω*) and *χ*″(*ω*) with *ω* as seen in Fig. 8(a,b), respectively. We thus note that a single spin in an external magnetic field is sufficient to reproduce this behaviour.

Any peak in the susceptibilities is usually associated with transitions that require interactions between the microscopic degrees of freedom (here spins). Instead of constructing a microscopic theory for interacting degrees of freedom for the present system, we approach it phenomenologically by taking cue from the experimental results and the physical picture drawn above, largely in the spirit of the well-known Curie-Weiss mean-field theory of ferromagnetism^31^. We start by assuming a temperature (*T*) dependent form for the *effective* damping Γ_*e*_(*T*):

15 $${{\rm{\Gamma }}}_{e}(T)={\rm{\Gamma }}+A{(T-{T}^{\ast })}^{2}+B{(T-{T}_{c})}^{2},$$

where *A* > 0 and *B* > 0 are phenomenological constants, T* and *T*_*c*_ are the two temperatures corresponding to the two peaks observed in Fig. 8 ^32^ (A more rigorous analysis would involve allowing Γ_*e*_ to have *both* real and imaginary parts as functions of *ω*, as will be enforced by the Kramer-Kronig relations^30^. We ignore such complications here). We also assume T* < *T*_*c*_, i.e., the peak at *T* = T* corresponds to the left peak in Fig. 8 that display reverse shift as *ω* rises. As discussed above, T* is the ordering temperature of an ordering transition that results from a competition between an ordering frequency-dependent external magnetic *h*(*ω*) and thermal energy *k*_*B*_*T* (*k*_*B*_ is the Boltzmann factor). We set

16 $${\langle h\rangle }_{t}^{2}={k}_{B}{T}^{\ast },$$

that yields T*, where the subscript *t* refers to the *temporal averaging h over a finite time t*. For a *ω*-dependent *h*, 〈*h*〉_*t*_ clearly decreases as *ω* increases: 〈*h*〉_*t*_ should be a monotonically decreasing function of *ω*. While the precise *ω*-dependence of 〈*h*〉_*t*_ should depend upon the functional form of *h*(*ω*), we assume 〈*h*〉_*t*_ ~ *C*/*ω*, where *C* is another phenomenological constant. This yields for the transition temperature T* ~ *C*/(*ω*^2^*k*_*B*_) that clearly decreases with *ω*, resulting into a reverse shift of the peak associated with T* (We ignore the issue of the singularity in T* as *ω* → 0. This is an artifact of the simple *ω*-dependence of 〈*h*〉_*t*_ chosen here. A more accurate knowledge of the *ω*-dependence of T* will rectify this and quantitatively change the form of T* as a function of *ω*. Nonetheless, the general trend should remain unchanged from here). This is consistent with the observed reverse shift with a rise in *ω* in the plots given in Fig. 8.

**Figure 9:**
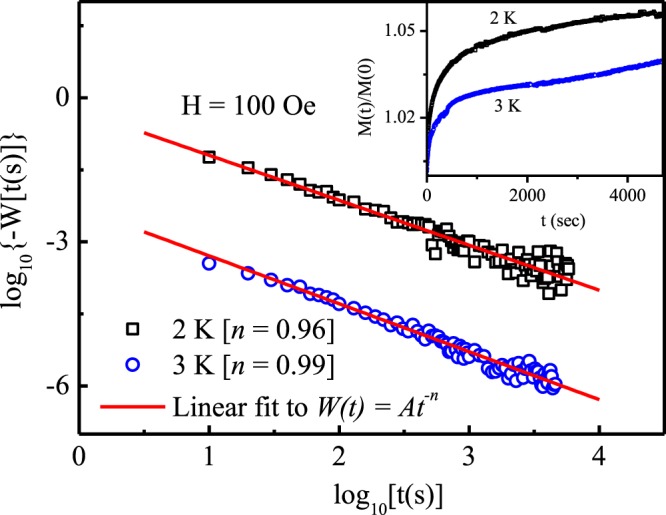
The log-log plot magnetic relaxation rate of Pr_2_Ni_0.95_Si_2.95_ vs time at different temperatures. Inset shows the the corresponding relaxations of normalized ZFC magnetization in 100 Oe field. The straight lines represent the linear fit to Eq. 17 and the slopes of the fitted straight lines are the values of exponent *n*.

To further confirm that the glassy state is originating from the magnetic clusters, we have performed magnetic relaxation behaviour at different temperatures (below T_f_) and analyzed the data by model proposed by Ulrich *et al*.^33^. This model applicable for a system consisting of interacting magnetic particles, suggest the rate of change of normalized remanent magnetization, *W*(*t*) = −(*d*/*dt*)[ln*m*(*t*)] (*m*(*t*) = *M*(*t*)/*M*(*t* = 0)), decays following the law,

17 $$W(t)=A{t}^{-n},\,t\ge {t}_{0}$$

where *A* is a constant prefactor, *n* is an density dependent exponent of temperature and *t*_0_ is crossover time after which the model is valid. The decay of *W*(*t*) and the values of *n* obtained for two different temperatures are shown in Fig. 9. At T = 3 K, which is in the vicinity of T_f_, *n* is close to 1 indicating quite strong intercluster interaction. Since *n* is a parameter which reflects the strength of magnetic interactions, the change in the value of *n* with temperature further confirm the growth of intercluster strength as freezing temperature is approached. This type of behaviour also confirm freezing of spin cluster rather than individual spin freezing.

**Figure 10:**
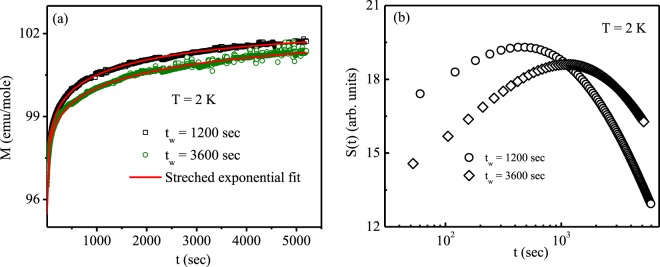
(**a**) Magnetic relaxation behaviours under ZFC protocol at 2 K along with stretched exponential fit [Eq. 18] and (**b**) the corresponding relaxation rate S(t) for different wait times, t_*w*_ = 1200 sec and 3600 sec for Pr_2_Ni_0.95_Si_2.95_.

Different types of glassy system are known to exhibit non-equilibrium dynamical behaviour below their freezing temperature. The systems remains in a non-equilibrium state and it exhibits slow magnetic relaxation dynamics over decades of time. In ZFC protocol, the magnetic relaxation behaviour have been measured by cooling the sample to a measurement temperature (T < T_f_) in zero magnetic field. After the lapse of certain time (*t*_*w*_) at the required temperature, small amount of external magnetic field (*μ*_0_*H* < *k*_*B*_*T*_*f*_) is applied and time dependence of magnetization [M(t)] is recorded. As seen from Fig. 10(a), the experimentally observed magnetic relaxation behaviour can be perfectly demonstrated by standard stretched exponential function of the form^2,34^,

18 $$M(t)={M}_{0}\pm {M}_{g}\,\exp \,(\,-\,{(\frac{t}{\tau })}^{\beta })$$

where *M*_0_ is the associated intrinsic magnetization of the system, *M*_*g*_ is related to magnetization fraction correspond to glassy magnetic phase, *τ* is called the relaxation time constant and *β* is known as stretching exponent. The value of *β* depends on the nature of energy barriers involves in the relaxation process. Since typical spin glass systems are classified with a distribution of energy scales, *β* values remains in the range 1 > *β* > 0.

As seen from Table 2, the obtained value of *τ* increases with increasing *t*_*w*_, indicating a stiffening of the spin relaxation with time. This clearly indicates the aging process in the system and also the signature of non-equilibrium dynamical behaviour. An inflection point occurs in the M(t) curve that shifts to higher observation time with increasing *t*_*w*_. The inflection point is best visualized as a peak in the magnetic viscosity curve, defined as,

19 $$S(t)=(1/H)\frac{dM(t)}{d({log}\,t)}$$

**Table 2:** Fitted parameters of the magnetic relaxation data of Pr_2_Ni_0.95_Si_2.95_ at 2 K for different wait times using Eq. 18.

*t*_*w*_ (sec)	*M*_0_ (emu/mole)	*M*_*g*_ (emu/mole)	*τ* (sec)	*β*
1200	102.58 (1)	7.15 (4)	520 (6)	0.32
3600	102.6 (1)	7.88 (1)	1163 (29)	0.32

The peak in S(t) curve shifts to higher observation time with increasing the wait time, *t*_*w*_, as shown in Fig. 10(b). This type of aging phenomena establish non equilibrium dynamical domain growth in Pr_2_Ni_0.95_Si_2.95_.

**Figure 11:**
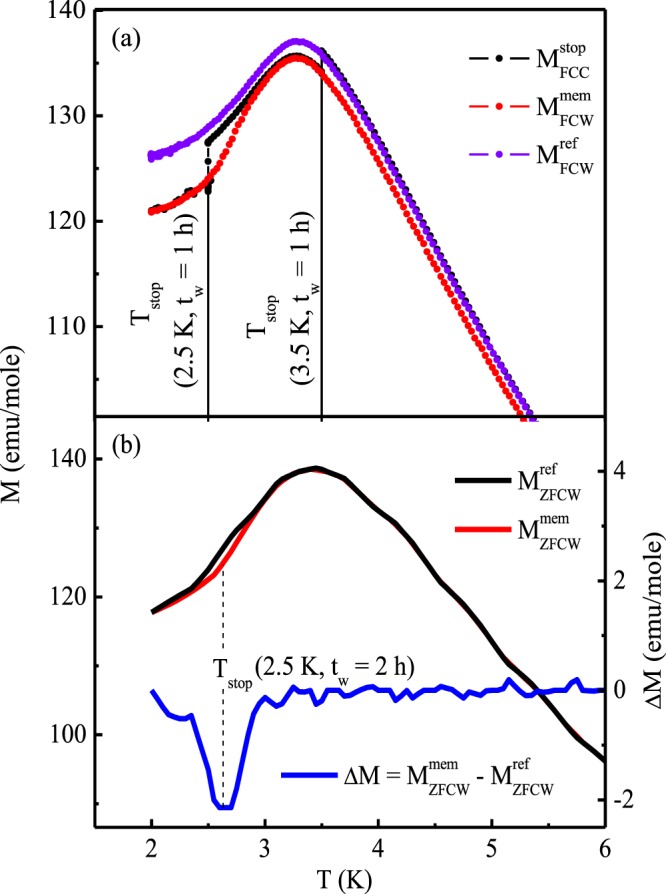
Magnetic memory effect of Pr_2_Ni_0.95_Si_2.95_ under (**a**) FC condition and (**b**) ZFC condition for 100 Oe applied field.

Magnetic memory effect for the compound has been measured in the thermal variation of the magnetization under both FC and ZFC process at an applied magnetic field value of 100 Oe. The whole measurement process has been carried out with constant temperature sweep rate, 1 K/min. In FC process, the sample was initially cooled down to the temperature 2 K, from the paramagnetic region (50 K) in the presence of magnetic field. During this cooling process the sample was “halted” at some stopping temperatures, T_stop_ = 3.5 K and 2.5 K of duration *t*_*w*_ = 1 h. After reaching each T_stop_, the magnetic field was removed and after the time lapse of *t*_*w*_ the same magnetic field was reapplied followed by resuming cooling process. This curve is defined as ${M}_{FCC}^{stop}$, as seen in the Fig. 11(a). The magnetization (${M}_{FCW}^{mem}$) was then measured in heating mode from 2 K to 50 K without any halt. It is clear from the Fig. 11(a) that, ${M}_{FCW}^{mem}$ shows an upturn beyond 2.5 K, *i.e*., it is trying to follow its previous history exhibiting magnetic memory effect. However, no such memory effect is observed in ${M}_{FCW}^{mem}$ curve beyond T_stop_ = 3.5 K, which is slightly higher that the spin freezing temperature for this system. This type of magnetic memory effect has also been observed in ZFC process [Fig. 11(b)]. ZFC memory effect is a more sensitive phenomena for spin glass systems and is forbidden in other types of non equilibrium systems. During this process, the sample was initially cooled down to the lowest temperature (2 K) in zero magnetic field with intermediate stop at T_stop_ = 2.5 K. The magnetization (${M}_{ZFCW}^{mem}$) measured during heating process when subtracted from conventional ZFC magnetization (${M}_{ZFCW}^{ref}$), the difference curve (${\rm{\Delta }}M={M}_{ZFCW}^{mem}-{M}_{ZFCW}^{ref}$) clearly exhibits a memory dip further confirming the spin glass state formation for this compound^35,36^.

**Figure 12:**
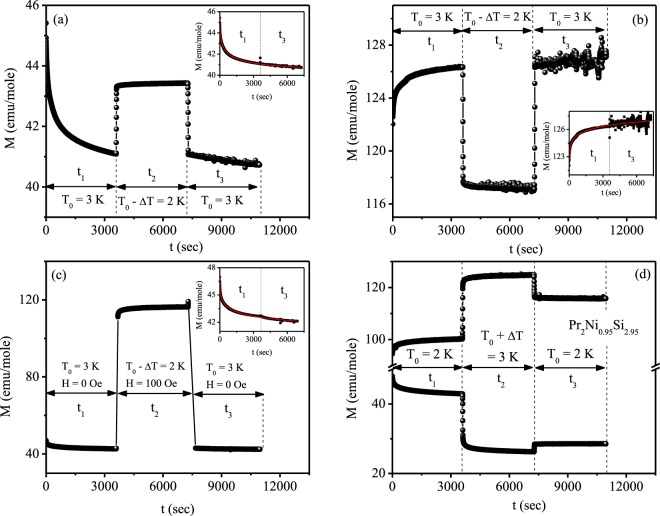
Magnetic relaxation of Pr_2_Ni_0.95_Si_2.95_ at 3 K for H = 100 Oe with temporary cooling to 2 K for the (**a**) FC method and (**b**) ZFC method. (**c**) Magnetic relaxation for the FC protocol at 3 K with opposite field changes during temporary cooling to 2 K. The insets show the relaxation data vs the total time spent at 3 K along with the fit (solid red line) using stretched exponential function [Eq. 18]. (**d**) Magnetic relaxation at 2 K with temporary heating to 3 K in both the ZFC and FC methods for 100 Oe applied magnetic field.

In a magnetic system with random magnetic anisotropy magnetic memory effect is best described by hierarchical model^37,38^ which favours multivalley spin structure on the free energy landscape, rather than droplet model^39,40^ that favours only one spin configuration at a particular temperature. Thus, symmetric relaxation behaviour is expected with respect to heating and cooling in droplet model while that should be asymmetric in Pr_2_Ni_0.95_Si_2.95_ system. To experimentally prove this, the relaxation behaviour is measured with both temporary cooling and heating using the protocol proposed by Sun *et al*.^35^. Figure 12(a,b) display isothermal magnetic relaxation in FC and ZFC protocols, respectively, at T_0_ = 3 K measured in 100 Oe with temporary cooling at T_0_ − ΔT = 2 K. Initially, M(t) was recorded for time, *t*_1_ = 1 h. At the end of *t*_1_, the temperature was quenched to 2 K and after time scale *t*_2_ = 1 h, the temperature was raised back to 3 K. Finally M(t) was measured at 3 K for *t*_3_ = 1 h. Figure 12(c) shows the magnetic relaxations at 3 K with switching on and off the magnetic field in consecutive time scale. M(t) was measured with 100 Oe magnetic field during *t*_2_, while M(t) were measured at zero field during *t*_1_ and *t*_3_. As seen from the figures, when the system is heated back to T_0_, the magnetizations are exactly retrieved to the same level it was before the temporary cooling processes. The M(t) during *t*_3_ is continuous to that during *t*_1_ and can be well described by single stretched exponential function of the form of Eq. 18. Thus, temporary cooling has no impact on the observed magnetic memory behaviour during relaxation process. However, the relaxation curve during *t*_3_ is totally different of that during *t*_1_ when the system is temporarily heated during the time interval *t*_2_ in both the ZFC and FC processes, as seen from Fig. 12(d). Thus relaxation behaviour cannot restore its previous history when it is temporarily heated to T_0_ + ΔT. This type of contradictory response upon cooling and heating is in accordance with the hierarchical model.

In conclusion, we report here the successful synthesis of a new ternary intermetallic compound Pr_2_Ni_0.95_Si_2.95_ that forms in single phase only by creating vacancies in Ni/Si site. The compound undergoes spin freezing behaviour below 3.3 K, characterized through meticulous studies on dc magnetization, heat capacity, ac magnetization & related dynamic scaling, several non-equilibrium dynamical behaviour. The primary interest in this material commence from the fact that Pr_2_Ni_0.95_Si_2.95_ is the only intermetallic compound that exhibits bidirectional frequency dependence of dynamical susceptibility - a fact hitherto not emphasized so far. The observation of unusual frequency dependence of the additional peak below the freezing temperature has been attributed due to the magnetic phase separation in this compound. Due to the presence of competing magnetic exchange interactions and atomic disorder, the magnetic ground state of the compound found to be consisting of macroscopically different magnetic phases, a magnetic glassy phase coexisting with nanoscopic non-correlated small magnetic clusters. The spin dynamics of such clusters has been argued to be responsible for such reverse frequency shift. Our proposed phenomenological model also reproduces the qualitative features observed in the low temperature ac susceptibility data. A full-fledged microscopic theory would be needed to calculate the form Γ_*e*_ that is used here in Eq. (15). Such an attempt, if successfully carried out, would also provide information about the phenomenological constants *A*, *B* and *C* introduced in the model. This remains a challenging theoretical task.

## Methods

The polycrystalline materials were synthesized by melting the calculated proper amount of constituent elements, *viz*., Pr, Ni and Si of high purity (>99.9%) in an arc furnace under inert (Ar) environment. Powder X-ray diffraction (XRD) measurements were carried out at different temperatures (15 K ≤ T ≤ 300 K) using Cu-K_*α*_ radiation on a TTRAX-III difractometer (M/s Rigaku, Japan). Structural characterization and the check of phase purity were performed by Rietveld refinement of XRD data using FULLPROF software package^18^. The scanning electron microscopy (SEM) measurements were carried out in the instrument EVO 18 (M/s Carl Zeiss, Germany) and the EDX measurements were performed in Element EDS system (M/s EDAX Inc., USA). Transmission electron microscopy (TEM) investigation was carried out using FEI, Tecnai G^2^ F30, ST microscope operating at 300 kV equipped with a Gatan Orius SC1000B CCD camera. Specimen for TEM was prepared by crashing the sample into power, dispersed in ethanol and mounting on a carbon coated copper grid. The compositional analysis was performed by energy dispersive X-ray spectroscopy (EDS, M/s EDAX Inc., USA) attachment on the Tecnai G^2^ F30. The dc magnetization was measured in a commercial SQUID VSM (M/s Quantum Design Inc., USA) & Ever Cool II VSM (M/s Quantum Design Inc., USA) and the ac susceptibility measurements were performed in a Ever Cool II VSM system (M/s Quantum Design Inc., USA). The heat capacity was measured by relaxation technique in a commercial PPMS system (M/s Quantum Design Inc., USA).

## References

[1] Ramirez, A. P. *Handbook of magnetic materials*, edited by Buschow, K. H. J. Vol. 13, ch. 4, (Elsevier, Amsterdam, 2001).

[2] Mydosh, J. A. *Spin glasses: An experimental introduction* (eds Taylor & Francis) ch. 3, (London, Washington, 1993).

[3] Matsunami D, Fujita A, Takenaka K, Kano M (2015). Giant barocaloric effect enhanced by the frustration of the antiferromagnetic phase in Mn_3_GaN. Nat. Mater..

[4] Babu GS (2011). New (Bi_1.88_Fe_0.12_) (Fe_1.42_Te_0.58_)O_6.87_ Pyrochlore with Spin-Glass Transition. Chem. Mater.

[5] Pakhira S, Mazumdar C, Ranganathan R, Giri S, Avdeev M (2016). Large magnetic cooling power involving frustrated antiferromagnetic spin-glass state in *R*_2_NiSi_3_ (*R* = Gd, Er). Phys. Rev. B.

[6] Pakhira S, Mazumdar C, Ranganathan R, Avdeev M (2017). Magnetic frustration induced large magnetocaloric effect in the absence of long range magnetic order. Sci. Rep..

[7] Pakhira S, Kundu AK, Mazumdar C, Ranganathan R (2018). Role of random magnetic anisotropy on the valence, magnetocaloric and resistivity properties in a hexagonal Sm_2_Ni_0.87_Si_2.87_ compound. J. Phys.: Condens. Matter.

[8] Gordon RA, Warren CJ, Alexander MG, DiSalvo FJ, Pöttgen R (1997). Substitution in Ce_2_TSi_3_ intermetallic compositions with T = (Cr, Mn, Fe, Co, or Ni)_*x*_(Pd or Au)_1−*x*_. J. Alloys Comp..

[9] Caignaert V (2009). A New Mixed-Valence Ferrite with a Cubic Structure, YBaFe_4_O_7_: Spin-Glass-Like Behavior. Chem. Mater.

[10] Benbow EM, Dalal NS, Latturner SE (2009). Spin Glass Behavior of Isolated, Geometrically Frustrated Tetrahedra of Iron Atoms in the Intermetallic La_21_Fe_8_Sn_7_C_12_. J. Am. Chem. Soc..

[11] Huo D, Sakurai J, Kuwai T, Isikawa Y, Lu Q (2001). Electric, magnetic, and thermal properties of Ce_2_NiGe_3_: A Kondo lattice compound showing spin glass behavior. Phys. Rev. B.

[12] Li DX (2003). ac susceptibility and magnetic relaxation of *R*_2_PdSi_3_ (*R* = Nd, Tb, and Dy). Phys. Rev. B.

[13] Pakhira S, Mazumdar C, Ranganathan R, Giri S (2018). Magnetic phase inhomogeneity in frustrated intermetallic compound Sm_2_Ni_0.87_Si_2.87_. J. Alloys Comp..

[14] Pakhira S, Mazumdar C, Ranganathan R, Giri S (2018). Chemical disorder driven reentrant spin cluster glass state formation and associated magnetocaloric properties of Nd_2_Ni_0.94_Si_2.94_. Phys. Chem. Chem. Phys..

[15] Pakhira S, Mazumdar C, Choudhury D, Ranganathan R, Giri S (2018). Observation of short range order driven large refrigerant capacity in chemically disordered single phase compound Dy_2_Ni_0.87_Si_2.95_. Phys. Chem. Chem. Phys..

[16] Majumdar S, Kumar MM, Sampathkumaran EV (1999). Magnetic behavior of a new compound, Gd_2_PdGe_3_. J. Alloys Comp..

[17] Pakhira S, Mazumdar C, Ranganathan R (2017). Low-field induced large magnetocaloric effect in Tm_2_Ni_0.93_Si_2.93_: influence of short-range magnetic correlation. J. Phys.: Condens. Matter.

[18] Rodrguez-Carvajal J (1993). Recent advances in magnetic structure determination by neutron powder diffraction. Physica B.

[19] Tang F (2011). Crystallographic superstructure in *R*_2_PdSi_3_ compounds (*R* = heavy rare earth). Phys. Rev. B.

[20] Hong CS, Kim WS, Chi, Hur NH, Choi YN (2002). Role of Rare Earth Ion in Spin Glass Behavior for R_0.7_Sr_1.3_MnO_4_. Chem. Mater.

[21] Poddar A, Bhowmik RN, Muthuselvam IP, Das N (2009). Evidence of disorder induced magnetic spin glass phase in Sr_2_FeMoO_6_ double perovskite. J. Appl. Phys..

[22] Hohenberg PC, Halperin BI (1977). Theory of dynamic critical phenomena. Rev. Mod. Phys..

[23] Lago J, Blundell SJ, Eguia A, Jansen M, Rojo T (2012). Three-dimensional Heisenberg spin-glass behavior in SrFe_0.90_Co_0.10_O_3.0_. Phys. Rev. B.

[24] Mori T, Mamiya H (2003). Dynamical properties of a crystalline rare-earth boron cluster spin-glass system. Phys. Rev. B.

[25] Souletie J, Tholence JL (1985). Critical slowing down in spin glasses and other glasses: Fulcher versus power law. Phys. Rev. B.

[26] Baker PJ (2005). Thermodynamic and magnetic properties of the layered triangular magnet NaNiO_2_. Phys. Rev. B.

[27] Sow C, Samal D, Anil Kumar PS, Bera AK, Yusuf SM (2012). Structural-modulation-driven low-temperature glassy behavior in SrRuO_3_. Phys. Rev. B.

[28] Nayek C (2016). Spin-glass state in nanoparticulate (La_0.7_Sr_0.3_MnO_3_)_1−*x*_(BaTiO_3_)_*x*_ solid solutions: Experimental and density-functional studies. Phys. Rev. B.

[29] Svedberg M, Majumdar S, Huhtinen H, Paturi P, Granroth S (2011). Optimization of Pr_0.9_Ca_0.1_MnO_3_ thin films and observation of coexisting spin-glass and ferromagnetic phases at low temperature. J. Phys.: Condens. Matter.

[30] Chaikin, P. M. & Lubensky, T. C. *Principles of Condensed Matter Physics* (Cambridge university press, 2000).

[31] Reif F (1965). Fundamentals of statistical and thermal physics.

[32] A *T*-dependent Γ_*e*_ is standard and can arise due to renormalisation of Γ_0_ by nonlinear interactions; see, *e.g*., P. C. Hohenberg and B. I. Halperin. *Rev. Mod. Phys*. **49**, 435, in the context of dynamic critical phenomena (1977).

[33] Ulrich M, Garca-Otero J, Rivas J, Bunde A (2003). Slow relaxation in ferromagnetic nanoparticles: Indication of spin-glass behavior. Phys. Rev. B..

[34] Binder K, Young AP (1986). Spin glasses: Experimental facts, theoretical concepts, and open questions. Rev. Mod. Phys..

[35] Sun Y, Salamon MB, Garnier K, Averback RS (2003). Memory effects in an interacting magnetic nanoparticle system. Phys. Rev. Lett..

[36] Sasaki M, Jönsson PE, Takayama H, Mamiya H (2005). Aging and memory effects in superparamagnets and superspin glasses. Phys. Rev. B.

[37] Dotsenko VS (1985). Fractal dynamics of spin glasses. J. Phys. C: solid State Phys..

[38] Vincent, E., Hammann, J., Ocio, M., Bouchaud, J. P. & Cugliandolo, L. F. *Complex Behaviour of Glassy Systems*, edited by Rubi, E. (Springer, Berlin, 1996).

[39] Fisher DS, Huse DA (1988). Nonequilibrium dynamics of spin glasses. Phys. Rev. B.

[40] Mcmillan WL (1984). Scaling theory of Ising spin glasses. J. Phys. C.

